# Physical Activity Environment and Japanese Adults’ Body Mass Index

**DOI:** 10.3390/ijerph15040596

**Published:** 2018-03-26

**Authors:** Mohammad Javad Koohsari, Andrew T. Kaczynski, Tomoya Hanibuchi, Ai Shibata, Kaori Ishii, Akitomo Yasunaga, Tomoki Nakaya, Koichiro Oka

**Affiliations:** 1 Faculty of Sport Sciences, Waseda University, Saitama 359-1192, Japan; ishiikaori@waseda.jp (K.I.); koka@waseda.jp (K.O.); 2 Behavioural Epidemiology Laboratory, Baker Heart and Diabetes Institute, Melbourne 3004, Australia; 3 Mary MacKillop Institute for Health Research, Australian Catholic University, Melbourne 3000, Australia; 4 Department of Health Promotion, Education, and Behavior, Arnold School of Public Health, University of South Carolina, Columbia, SC 29229, USA; atkaczyn@mailbox.sc.edu; 5 Prevention Research Center, University of South Carolina, Columbia, SC 29229, USA; 6 School of International Liberal Studies, Chukyo University, Nagoya 466-8666, Japan; hanibuchi@gmail.com; 7 Faculty of Health and Sport Sciences, University of Tsukuba, Tsukuba 305-8574, Japan; shibata.ai.ga@u.tsukuba.ac.jp; 8 Faculty of Liberal Arts and Sciences, Bunka Gakuen University, Tokyo 151-8523, Japan; yasunaga@bunka.ac.jp; 9 Department of Geography and Institute of Disaster Mitigation for Urban Cultural Heritage, Ritsumeikan University, Kyoto 603-8577, Japan; nakaya@lt.ritsumei.ac.jp

**Keywords:** urban design, neighbourhood, weight, Asia, active behaviour, urban form

## Abstract

Evidence about the impacts of the physical activity environment on adults’ weight in the context of Asian countries is scarce. Likewise, no study exists in Asia examining whether Walk Score*^®^*—a free online walkability tool—is related to obesity. This study aimed to examine associations between multiple physical activity environment measures and Walk Score*^®^* ratings with Japanese adults’ body mass index (BMI). Data from 1073 adults in the Healthy Built Environment in Japan study were used. In 2011, participants reported their height and weight. Environmental attributes, including population density, intersection density, density of physical activity facilities, access to public transportation, and availability of sidewalks, were calculated using Geographic Information Systems. Walk Scores*^®^* ratings were obtained from the website. Multiple linear regression analysis was conducted to examine the association between each environmental attribute and BMI. Adjusting for covariates, all physical activity environmental attributes were negatively associated with BMI. Similarly, an increase of one standard deviation of Walk Score*^®^* was associated with a 0.29 (95% confidence interval (CI) of −0.49–−0.09) decrease in BMI. An activity-friendly built environment was associated with lower adults’ BMI in Japan. Investing in healthy community design may positively impact weight status in non-Western contexts.

## 1. Introduction

Despite considerable focus and prevention efforts, obesity rates have dramatically risen across the world [1]. For example, the prevalence of obesity has increased among USA adults since 1980: the rate of obesity was 35.0% among men and 40.4% among women in 2013–2014 [2]. In Canada, the proportion of obese persons was 26.7% in 2015, an increase from 23.1% in 2004 [3]. In Japan, the prevalence of overweight in males has almost doubled over the last 35 years [4]. An epidemic of obesity has also been developing in many Asian countries, where traditionally obesity was not a problem [5,6]. ‘Healthy Japan 21’, the official plan developed by the Japanese Ministry of Health, Labour and Welfare to enhance the nation’s health, has acknowledged the obesity issue through targeting an “increase in percentage of individuals maintaining ideal body weight” [7]. Therefore, there is a need for a holistic multi-level approach to tackle obesity worldwide [8].

There has been a growing interest in the role of the built environment—especially food and physical activity environments—on obesity [9,10,11]. The food environment refers to the availability of and access to healthy foods such as fruits and vegetables in communities [12]. Many studies have found food environment to be associated with healthy dietary intake and weight status [13,14]. The physical activity environment refers to neighbourhood attributes such as residential density, intersection density, availability of local shops, access to parks, and safety from crime which support residents’ recreational and transportation-related physical activity [15,16,17,18]. Many studies have also examined associations between the physical activity environment and weight status [19,20,21]. For example, a recent study conducted in the UK found strong associations between better availability of physical activity facilities (e.g., gyms, swimming pools, and playing fields) and lower adults’ obesity [22]. Another study in the USA found a composite measure of neighbourhood walkability (including population density, housing type, median home age, and commuting patterns) to be negatively associated with adults’ body mass index (BMI) [23].

However, this body of research is limited in two important ways. First, the majority of previous studies examining associations between the physical activity environment and obesity have been conducted in Western countries. For example, a systematic review of physical environment determinants of adults’ weight status found no studies conducted in Asian countries on this topic [21]. Many cities in Asian countries such as Japan have different environmental attributes compared with those in Western countries, such as higher population densities and efficient public transportation systems [24,25]. Several studies have examined associations between neighbourhood environmental attributes and adults’ active and sedentary behaviours in Japan [26,27], Hong Kong [28], South Korea [29], and China [30]. However, evidence about the impacts of such attributes on adults’ weight in the context of Asian countries is scarce.

Second, previous studies have mainly used geographic information systems (GIS) to calculate physical activity environmental measures [31]. This method allows for precise calculation of the built environment attributes, but can necessitate specialized GIS expertise and access to detailed spatial data [32]. For example, common GIS-based indices of neighbourhood require parcel-level data on land uses and residential density [33]. However, such spatial data are often unavailable or difficult to obtain not only in low-income, but also high-income countries [34,35]. Thus, online freely-available tools to conceptualise the physical environment can be important for practitioners and policy makers. Walk Score*^®^* is a free, online commercial tool, which calculates the walkability of a location (www.walkscore.com). Several validation studies reported associations between Walk Score*^®^* ratings and neighbourhood walkability attributes in the USA [36], Canada [37], and more recently in Japan [38]. An association between Walk Score*^®^* and obesity measures was also reported by few studies in the USA [39] and France [40]. However, as of yet, no study exists in the context of Asia examining whether Walk Score*^®^* is related to obesity.

Therefore, the purposes of the current study were to: (1) examine associations between the physical activity environment calculated by GIS and BMI among Japanese adults; and (2) to test whether Walk Score*^®^*, a freely accessible composite measure of walkability, is associated with Japanese adults’ BMI.

## 2. Materials and Methods

### 2.1. Study Setting and Sample

This cross-sectional study was part of the Healthy Built Environment in Japan (HEBEJ) project, a prospective investigation of the influence of built environment attributes on health behaviours and outcomes in Japan. HEBEJ focused on middle-to-older aged adults, because age-related functionality declines and other associated health problems usually begin at this life stage [41].

**Figure 1:**
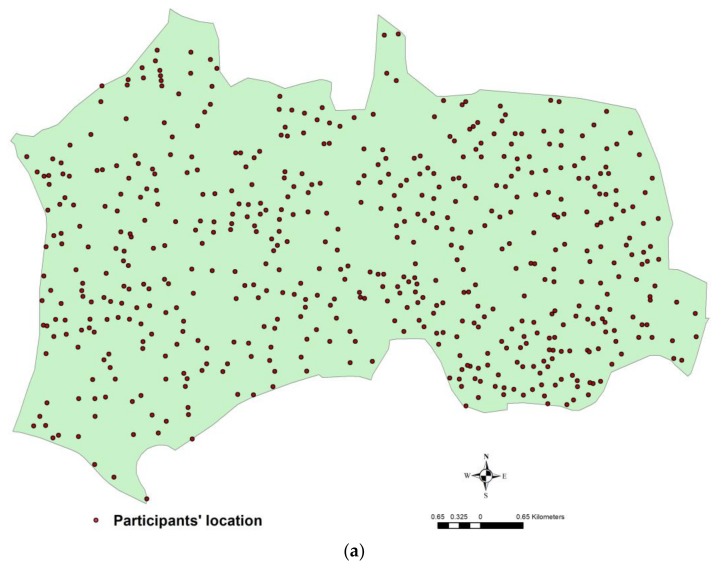

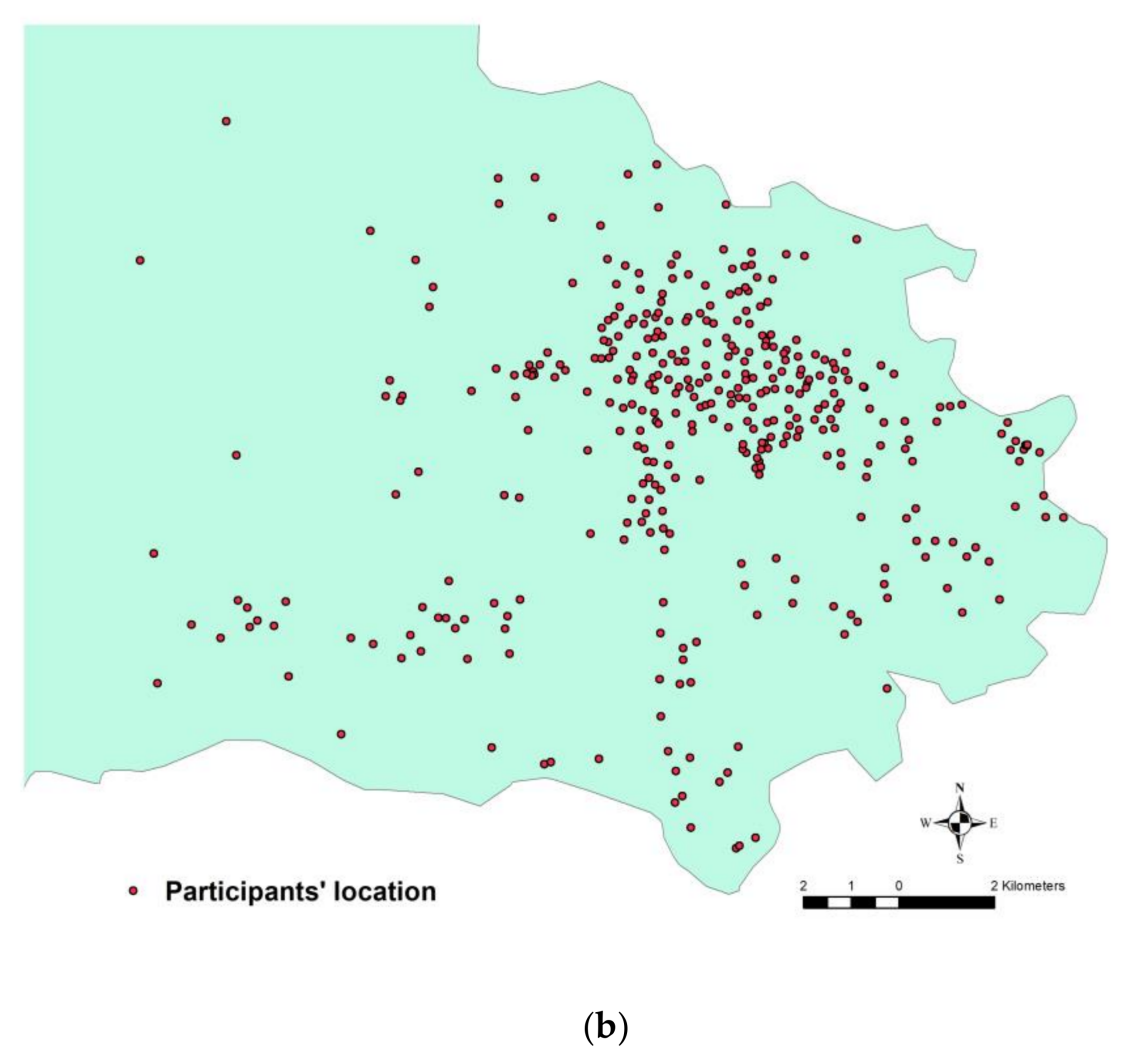
Sample location across (**a**) Nerima Ward and (**b**) Kanuma City.

Data were collected in 2011 from residents who lived in two areas (Nerima Ward and Kanuma City) in Japan. Nerima Ward is part of the Tokyo Metropolitan area, and Kanuma City is in a more rural area located approximately 120 km from Tokyo. Guided by the methodology of the International Physical Activity and Environment Network (IPEN) studies [35], participants were selected from high walkable (Nerima Ward) and low walkable areas (Kanuma City). This procedure assures variability in the relevant built environment measures [42]. A total of 1500 middle-to-older aged residents (40–69 years old) were randomly selected from the government registry of residential addresses across each city and were invited to participate in the study. Of these, 1076 residents agreed to participate and completed the postal survey (response rate: 35.9%). Figure 1a,b show the locations of respondents in Nerima Ward and Kanuma City, respectively. All participants provided written informed consent. The survey and its linkage with environmental measures were approved by the Institutional Ethics Committee of Waseda University (2010-238).

### 2.2. Measures

Body mass index: BMI (kg/m^2^) was calculated using self-reported height and weight. BMI was used as a continuous variable in this study.

Physical activity environmental attributes: five physical activity environmental attributes, including population density, intersection density, density of physical activity facilities, access to public transportation, and availability of sidewalks, were included in this study. These variables were defined as follows: (a) population density (number of residents per km^2^); (b) intersection density (number of three-way or more intersections per km^2^); (c) density of physical activity facilities (number of parks, and gym, fitness, and sport facilities per km^2^); (d) access to public transportation (number of train stations and bus stops per km^2^); (e) availability of sidewalks (the length of roads with sidewalks in km per km^2^). These attributes were chosen based on previous studies showing their associations with walking behaviours [15,16]. GIS data layers were obtained from the Environmental Systems Research Institute (ESRI) Japan data 2011 and ArcGIS software (ESRI Japan Corp., Tokyo, Japan) was used to calculate these measures within an 800 m (half mile) buffer around participants’ geocoded residential address. The choice of 800 m buffer was similar to previous studies examining associations between environmental attributes and active behaviours among middle-to-older aged adults [43,44].

Walk Score*^®^*: the Walk Score*^®^* algorithm first assigns a raw score to each location based on access to a variety of destinations such as schools, banks, and local shops within a 1.6 km (1 mile) network-distance of that location. These raw scores are then normalised from 0 to 100 according to the intersection density and block length around that location [45]. Locations with higher Walk Score*^®^* values provide more opportunities for their residents to walk (e.g., more local destinations and well-connected streets). In this study, two independent project members in 2016 entered each participant’s residential address into the Walk Score*^®^* website (www.walkscore.com) and extracted the values. Any discrepancy between two members was checked by the first author.

Covariates: the following sociodemographic characteristics were reported by participants: age, gender, employment status (employed, unemployed), education attainment (tertiary or higher, below tertiary), marital status (single, couple), and household income (<¥5,000,000; ≥¥5,000,000). Participants were also asked about their smoking habits (smoker, non-smoker) and self-rated health (very healthy, healthy, not healthy, not healthy at all). We included vigorous physical activity as a covariate to adjust for those physical activities which may be less related to the built environment. Vigorous physical activity (min per week) was assessed with the International Physical Activity Questionnaire (IPAQ) [46]. Participants’ leisure-time sedentary behaviours were obtained by aggregating seven types of self-reported behaviours: television or video watching; computer and internet use for leisure; video game use; reading; sitting and talking with friends or listening to music; talking on the telephone; and driving or riding in a car for leisure. The reliability and validity of these sedentary behaviour items has been previously reported [47].

### 2.3. Analysis

Descriptive analyses were used to calculate means and standard deviations for all study variables. Multiple linear regression analysis was conducted to examine the association between each physical activity environmental attribute and Walk Score*^®^* with BMI. All environmental attributes and Walk Score*^®^* were standardized to enable comparison. Six individual regression models were developed for each environmental attribute and Walk Score*^®^* with each adjusted for sociodemographic, smoking, self-rated health, physical activity, and sedentary behaviour variables. All analyses were conducted using Stata 15.0 (Stata Corp, College Station, TX, USA) and the level of significance was set at *p* < 0.05.

## 3. Results

After excluding those with missing BMI values (*n* = 3), data from 1073 participants were included in this study. Table 1 shows the characteristics of the sample. The mean age was 55.6 years, almost half (48.2%) were female, more than two-thirds (73%) were employed, more than half (52.8%) completed a tertiary or higher education, more than 80% were part of a couple, and almost half (48.5%) had an annual household income ≥¥5,000,000. The mean BMI was 23.0 kg/m^2^ with a median of 22.8 kg/m^2^.

Table 2 shows the participants’ physical activity environmental attributes and Walk Score*^®^* ratings. The means of population density and intersection density were 9486.9 residents and 353.4 intersections per square km^2^, respectively. Participants had access to about 1.5 physical activity facilities, 12.6 public transportation stations, and 12.2 km of roads with sidewalks per km^2^. The mean Walk Score*^®^* was 62.1 (standard deviation (SD) = 27.5) with a median of 73.0. There were also significant positive correlations between Walk Score*^®^* and population density (*r* = 0.72), intersection density (*r* = 0.81), density of physical activity facilities (*r* = 0.50), access to public transportation (*r* = 0.76), and availability of sidewalks (*r* = 0.76).

Adjusting for covariates, all physical activity environmental attributes were negatively associated with BMI (Table 3). A one standard deviation increase in population density, intersection density, density of physical activity facilities, access to public transportation, and availability of sidewalks, was associated with a 0.34, 0.26, 0.25, 0.22, and 0.38 decrease in BMI, respectively. An increase of one standard deviation of Walk Score*^®^* was also associated with a 0.29 decrease in BMI. The *r*-squared values from the linear regression indicate that about 9%, 8%, 8%, 8%, 9%, and 8% of variation in the BMI can be explained by population density, intersection density, density of physical activity facilities, access to public transportation, availability of sidewalks, and Walk Score*^®^*, respectively.

## 4. Discussion

This was the first study, to our knowledge, to examine associations between objectively-measured physical activity environmental attributes and Japanese adults’ BMI. We found all built environment attributes to be significantly associated with adults’ weight status—adults who lived in more-populated areas with well-connected streets and available sidewalks—and higher number of physical activity facilities and public transport stations nearby have significantly lower BMI values. These findings are similar with some previous studies conducted in Western countries, which found associations between walkable neighbourhood attributes and obesity [48,49,50,51,52]. For example, a recent study in Canada found higher residential density and availability of local destinations to be negatively associated with adults’ obesity [50]. Similarly, a longitudinal study conducted in the USA found increases in population and destination densities to be related to lower adult BMI [51]. One of the few (if not the only) prior studies on adults conducted in Asia found higher land use mix to be associated with lower BMI among Chinese adults [53]. Our findings provide evidence about the role of the physical activity environment in adults’ obesity prevention in Japan, where, similar with other countries, the prevalence of obesity and non-communicable diseases is growing [4]. In addition, most previous research has primarily been conducted in Western countries, which have lower levels of environmental attributes such as population density and availability of destinations. Therefore, it is not clear how such environmental attributes at the higher end of the spectrum can influence obesity. This study adds to the broader literature by testing effects on BMI in an Asian context with extreme levels of density and other environmental factors.

There may be several explanations for the negative associations between walkable neighbourhood attributes and BMI. Studies have shown that people who lived in well-connected areas having a variety of destinations nearby were more likely to walk, especially for transport [54,55]. Thus, such environmental attributes may influence BMI through the accrued physical activity [56,57]. Sedentary behaviour—a distinct behaviour with adverse health consequences [58]—is another pathway through which neighbourhood attributes may influence obesity. Indeed, several built environment attributes, such as street connectivity and population density, have been found to be associated with sedentary behaviours [59,60]. Finally, social capital and mental health may act as mediators between neighbourhood attributes and BMI [61,62]. Future studies should further investigate conceptual and empirical pathways in the relationship between physical activity environmental attributes and BMI in the context of Asia.

Previous studies conducted in Western countries found Walk Score*^®^* to be associated with adults’ BMI [39,40,63]. For instance, a recent study in France found a one unit increase in Walk Score*^®^* to be significantly associated with a 0.01 decrease in BMI [40]. Another longitudinal study in the USA found moving to a location with a higher Walk Score*^®^* to be associated with a reduction in adults’ BMI [39]. Our study confirmed these results and extended them by examining Walk Score*^®^* in relation to adults’ BMI in a non-Western context. These findings provide evidence for public health and urban design practitioners and policy makers in Asia on using Walk Score*^®^*, as a freely-accessible tool, to target possible areas for environmental obesity interventions. Since the association between Walk Score*^®^* and BMI may not always be linear, future studies should explore what levels of Walk Score*^®^* are necessary to influence obesity measures.

This study has some limitations. Our self-reported measures of height and weight and vigorous physical activity may be subject to self-report bias. As a cross-sectional study, we were also unable to determine causality. Our results may also be subject to non-response bias and our sample was not entirely representative of Japanese adults (i.e., only included adults 40 to 69 years and more employed). We measured physical activity environmental attributes only around participants’ homes. However, people can walk at other destinations such as workplaces, schools, and shops, and future studies should examine the effects of environmental attributes measured in a variety of locations on BMI. As well, the Walk Score*^®^* was based on destinations within a one-mile network buffer around participants, while physical activity environmental attributes were calculated within an 800 m buffer around each address. However, this difference is mitigated substantially because the Walk Score*^®^* algorithm uses a distance decay function to calculate scores, such that those destinations within 800 m are assigned much higher weights compared to those beyond 800 m [37]. Therefore, using the same one-mile buffer to calculate environmental attributes may have overestimated these attributes for areas beyond 800 m. Nonetheless, the use of circular buffers instead of network-based buffers in calculating environmental attributes was one of the limitations in this study. In addition, this study focused merely on the physical activity environment. Future studies can examine the joint impacts of physical activity and food environments on obesity. Furthermore, we did not include all relevant physical activity environmental attributes such as safety from traffic and crime [64,65]. The strength of this study is that data were collected from areas with diverse built environments, which added to variability in our exposure ranges within this novel setting.

## 5. Conclusions

While the role of physical activity environmental attributes on adults’ obesity has received much attention in the context of Western countries, there is limited evidence to date on this topic in Asia. Our study showed an activity-friendly built environment is associated with lower adults’ BMI in a Japanese context. This study also found, for the first time, that Walk Score*^®^*—a free online walkability tool—was associated with obesity in a non-Western context. Investing in healthy community design may positively impact weight status in non-Western contexts.

## Figures and Tables

**Table 1:** Characteristics of study participants (*n* = 1073).

Variable	Mean (SD) or *n* (%)
Age (years)	55.6 (8.4)
Gender	
Women	517 (48.2)
Men	556 (51.8)
Employment status	
Employed	783 (73.0)
Unemployed	281 (26.2)
Educational attainment	
Tertiary or higher	567 (52.8)
Below tertiary	505 (47.1)
Marital status	
Single	167 (15.6)
**Couple**	903 (84.2)
Household income (per annum)	
<¥5,000,000	532 (49.6)
≥¥5,000,000	520 (48.5)
Smoking habits	
Smoker	222 (20.7)
Non-smoker	851 (79.3)
Self-rated health	
Very healthy	87 (8.1)
Healthy	816 (76.0)
Not healthy	127 (11.8)
Not healthy at all	25 (2.3)
Vigorous physical activity (min/wk)	32.2 (71.1)
Leisure-time sedentary behaviours (min/wk)	267.1 (167.6)
BMI (kg/m^2^)	23.0 (3.2)

SD: standard deviation; wk: week; BMI: body mass index.

**Table 2:** Participants’ physical activity environmental attributes.

Environmental Attributes	Mean	SD
Population density ^a^	9486.9	8118.2
Intersection density ^b^	353.4	230.9
Density of physical activity facilities ^c^	1.6	2.2
Access to public transportation ^d^	12.6	7.1
Availability of sidewalks ^e^	12.2	7.7
Walk Score*^®^*	62.1	27.5

^a^ the number of residents per km^2^; ^b^ the number of intersections per square km^2^; ^c^ the number of parks, and gyms, fitness, and sport facilities per km^2^; ^d^ the number of train stations and bus stops per km^2^; ^e^ km of roads with sidewalks per km^2^.

**Table 3:** Associations of physical activity environmental attributes and Walk Score*^®^* with BMI.

Environmental Attributes	β	95% CI	*p*-Value
Population density	−0.34	−0.54, −0.15	0.00
Intersection density	−0.26	−0.46, −0.06	0.01
Density of physical activity facilities	−0.25	−0.45, −0.06	0.01
Access to public transportation	−0.22	−0.41, −0.02	0.03
Availability of sidewalks	−0.38	−0.57, −0.18	0.00
Walk Score*^®^*	−0.29	−0.49, −0.09	0.00

Note: All models adjusted for age, gender, employment status, educational attainment, marital status, household income, smoking habits, self-rated health, vigorous physical activity, leisure-time sedentary behaviour; CI: confidence interval.
